# Adjuvant trastuzumab for triple-positive breast cancer with chronic renal failure: A case report and review of literature

**DOI:** 10.1097/MD.0000000000036278

**Published:** 2024-01-05

**Authors:** Wen En, Yuming Long

**Affiliations:** a Department Of Oncology, The first people’s hospital of Neijiang, Neijiang, Sichuan, China.

**Keywords:** endocrine therapies, hemodialysis, renal failure, trastuzumab, triple-positive breast cancer

## Abstract

**Rationale:**

Although the occurrence of combined renal insufficiency among patients with breast cancer is even rarer, it poses a significant challenge in the treatment of these patients. Treating such patients often requires both targeted and endocrine therapies. However, oncologists lack evidence-based guidelines for managing renal function in patients with renal insufficiency.

**Patients concern:**

A 56-year-old menopausal female with a history of renal failure was diagnosed with triple-positive breast cancer and administered endocrine therapy and targeted therapy associated with hemodialysis after surgery.

**Outcomes:**

Under the premise of regular dialysis, the patient successfully completed endocrine therapy and targeted therapy for 1 year.

**Discussion:**

Patients with advanced triple-positive breast cancer, including those undergoing hemodialysis, require a combination of anti-human epidermal growth factor receptor-2 and endocrine therapies, The side effects of these 2 treatment methods are worth considering in patients with renal insufficiency.

**Conclusion:**

We report a case of triple-positive breast cancer in a patient undergoing hemodialysis. There was no difference in the treatment approach between patients with and without normal renal function.

## 1. Introduction

Standardized dialysis treatment has been shown to extend the survival time of patients with renal failure,^[1]^ and its efficacy is more obvious in.^[2]^ Approximately 10% of breast cancer patients are diagnosed with triple-positive breast cancer.^[3]^ Although the occurrence of combined renal insufficiency among these patients is even rarer, it poses a significant challenge for breast cancer treatment. Treating such patients often requires both targeted and endocrine therapies; however, oncologists lack evidence-based guidelines for managing renal function in patients with renal insufficiency. Meanwhile, kidney physicians find it challenging to provide adequate oncological treatment to such patients.

## 2. Case presentation and methodology

### 2.1. Case presentation

**Figure 1.**
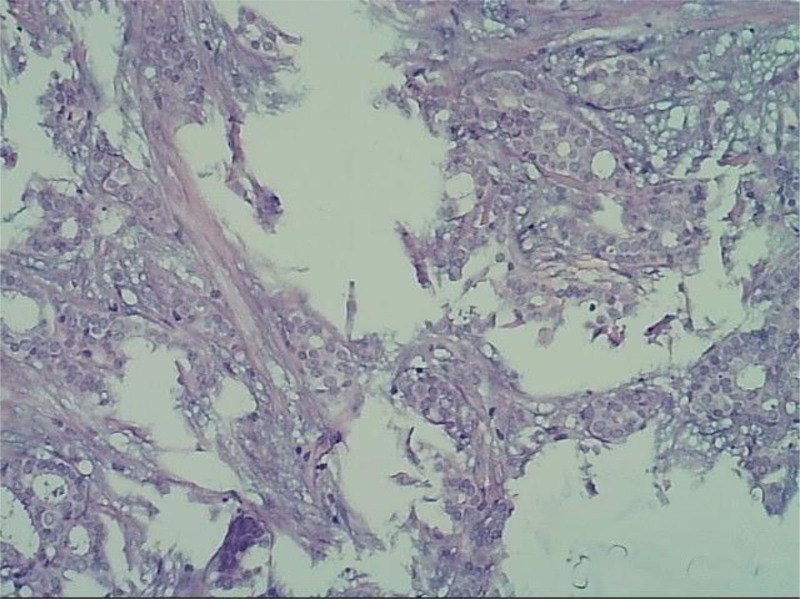
Pathological biopsy hematoxylin-eosin staining.

**Figure 2.**
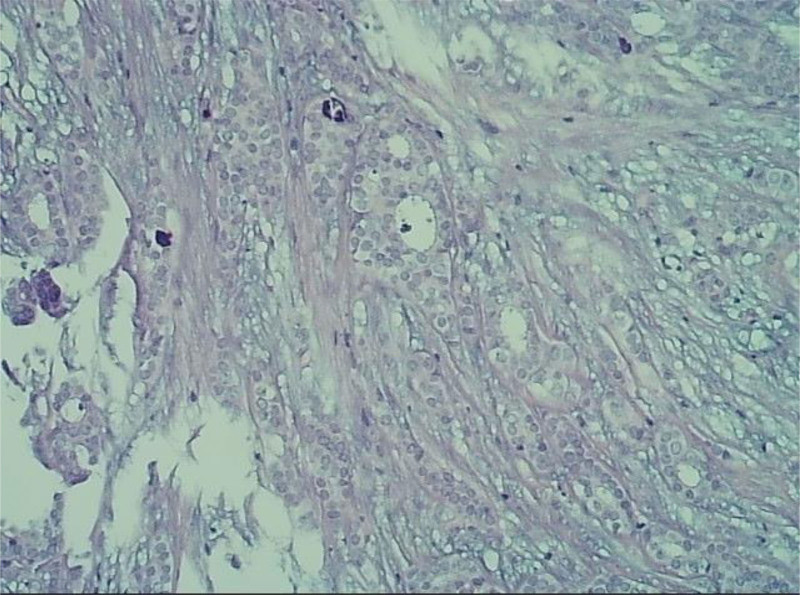
Pathological biopsy hematoxylin-eosin staining.

**Figure 3.**
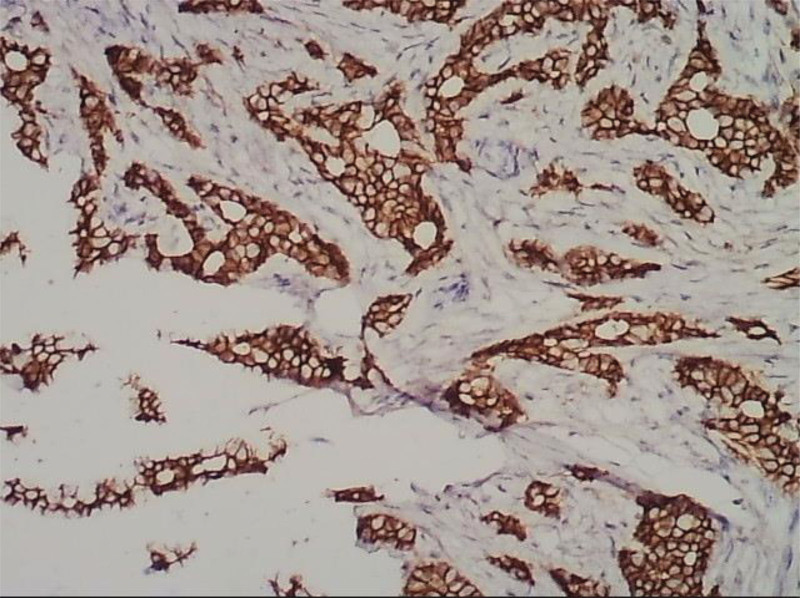
HER-2 (+++) immunohistochemical. HER-2 = human epidermal growth factor receptor-2.

A 56-year-old menopausal female had a mass of 1.5 * 1.0 cm at around 11 o’clock, as revealed by breast palpation in November 2021. Mild tenderness was present, but no local redness, itching, skin changes, or inverted nipple was observed. Color ultrasound revealed a weak-echo nodule at the 11 o’clock position of the left breast, classified as a BI-Rads4b nodule. After surgical resection, pathological examination confirmed the presence of invasive cancer, specifically invasive tubular cancer (Figs. 1 and 2). The specimen was subsequently sent to the West China Hospital for further pathological consultation: estrogen receptor (+,90%), progesterone receptor (+,90%), human epidermal growth factor receptor-2 (HER-2) (+++), CK-H(+++), E-Cad (+++), CK5/6 (−), Ki-67 (+,10%), and further treatment was recommended. Moreover, the patient had a history of chronic kidney failure for 7 years, necessitating regular hemodialysis twice a week on Tuesdays and Saturdays, starting from December 9, 2021. Breast cancer-modified radical surgery was performed at our hospital under general anesthesia on December 28, 2021. Postoperative examination revealed altered adenopathy in the resected breast tissue with small amounts of papillary composition in the catheter. Furthermore, partial catheter dilation with endocrine and chronic inflammatory cell infiltration was observed around the catheter. Focal calcification, angiogenesis, heme deposition, bleeding, chronic inflammatory cell infiltration, and reactions from tissue cells and multicore macrocells were observed. No residual cancer was identified, and no tumors were found in the basal margins (top, bottom, inner, and outer), nipple, or skin. None of the 14 axillary lymph nodes showed metastasis (0 of 14). The postoperative diagnosis was left breast cancer [T1N0M0, estrogen receptor (+), progesterone receptor (+), HER-2 (+) (Fig. 3).

### 2.2. Methods

Following the 2022 the National Comprehensive Cancer Network^[4]^ and Chinese Society of Clinical Oncology guidelines, we initiated letrozole at a dose of 2.5 mg qd as endocrine therapy, 3 weeks after surgery. For targeted therapy, trastuzumab was administered at a dose of 8 mg/kg (maintenance therapy, 6 mg/kg), 3 times per week. To ensure treatment efficacy, hemodialysis was conducted twice weekly on Tuesdays and Saturdays. Trastuzumab was typically administered on the second day after dialysis, and it was continued for 1 year, with intermittent treatment in September 2022 because of the outbreak of Corona Virus Disease 2019. Subsequently, the targeted therapy was resumed, starting with an initial dose of 8 mg/kg trastuzumab, followed by maintenance therapy at 6 mg/kg.

## 3. Outcomes

After 1 year of targeted therapy with trastuzumab, the patients showed no signs of tumor recurrence or metastases during follow-up while receiving letrozole endocrine therapy. The frequency of hemodialysis was consistent twice weekly. There were no notable instances of major cardiac dysfunction, significant deterioration in renal function, poor glycemic control, or any other complications.

## 4. Discussion

Patients with renal insufficiency are at a higher risk of developing malignant neoplasms than those without. However, the use of various antitumor drugs is limited because of renal failure. Many chemotherapy drugs exhibit hepatorenal side effects; however, with appropriate coordination between dialysis and chemotherapy drug schedules, it is still feasible to safely complete chemotherapy. Targeted and endocrine therapies typically entail milder toxic side effects than cytotoxic chemotherapeutic drugs, thereby reducing concerns regarding their impact on dialysis and associated toxic side effects.

The primary toxic side effects of trastuzumab include cardiac dysfunction, bone marrow suppression, and allergic reactions. Renal impairment is rarely reported, and there are limited reports on the use of trastuzumab in patients with renal failure.^[5,6]^ Gaertner et al reported a case in which a patient with invasive ductal breast carcinoma undergoing hemodialysis received 6 cycles of neoadjuvant docetaxel, carboplatin, trastuzumab, and pertuzumab. Due to a decline in left ventricular ejection fraction, the patient continued with trastuzumab to complete 1 year of HER2-targeted therapy.^[7]^ Additionally, Matsumoto et al presented a case of breast cancer with liver metastasis in a patient who underwent hemodialysis. The patient received multiple courses of chemotherapy and was treated with trastuzumab emtansine, trastuzumab, and pertuzumab, and achieved an overall survival of 25 months.^[8]^ Falcón González presented a case study involving a 73-year-old female patient with early breast cancer and multiple complications who was receiving hemodialysis. The patient developed bone metastases after conservative breast surgery and axillary dissection. Trastuzumab was administered as a first-line therapy, resulting in a long period of disease stability. However, the tumor subsequently recurred, prompting the initiation of ado-trastuzumab emtansine as second-line therapy, which led to an almost complete response.^[9]^ Gori described a case of a breast cancer patient with end-stage renal disease undergoing hemodialysis. The patient received a combination of paclitaxel and cyclophosphamide chemotherapy, followed by radiotherapy and trastuzumab. The data demonstrated that the pharmacokinetics of trastuzumab were comparable between patients undergoing hemodialysis and those without renal impairment.^[10]^ Similarly, Piacentini reported the case of a breast cancer patient with liver metastasis and renal failure. The patient initially received a first-line treatment with anastrozole and trastuzumab. When the liver disease progressed, the patient received a combination therapy involving letrozole and lapatinib, resulting in effective disease control.^[11]^

## 5. Conclusion

According to the National Comprehensive Cancer Network and Chinese Society of Clinical Oncology guidelines, patients with advanced triple-positive breast cancer, including those undergoing hemodialysis, require a combination of anti-HER-2 and endocrine therapies. Adjuvant postoperative therapy with trastuzumab and letrozole is also recommended. There was no difference in the treatment approach between patients with and without normal renal function.

## Author contributions

**Methodology:** Wen En.

**Resources:** Wen En, Yuming Long.

**Writing – original draft:** Wen En.

**Writing – review & editing:** Yuming Long.
